# The Effectiveness of Motivational counseling in improving Psychological vitality of Teachers in Dhofar Region Schools - Sultanate of Oman

**DOI:** 10.1192/j.eurpsy.2022.1792

**Published:** 2022-09-01

**Authors:** N. Abdelrasheed, M. Khalaf

**Affiliations:** 1 Dhofar University, Education, Salalah, Oman; 2 Minia University, Mental Hygiene, Minia, Egypt; 3 Minia University, Educational Psychology, Minia, Egypt; 4 Sultan Qaboos University, Psychology, Muscat, Oman

**Keywords:** Counseling program, motivational counseling, psychological vitality, School teachers

## Abstract

**Introduction:**

Psychological vitality has a significant relationship with self-realization, mental health, positive emotions and self-motivation, life satisfaction, and optimism. Additionally, it reduces the possibility of exposure to anxiety, depression and stress. In the same line, work pressures drive teachers to suffer from some negative emotions such as anger and lack of motivation, which in turn influence their vitality, social relations with colleagues.

**Objectives:**

The present study designed a motivational counseling-based program to improve psychological vitality among teachers in Dhofar region schools

**Methods:**

Participants were 60 teachers obtained the lowest degrees on the psychological vitality questionnaire. They were divided randomly into two groups: experimental and control. Qusai-experimental method with two groups design was adopted. The given program consists of 18 counseling sessions at the rate of 3 sessions per week ranging from 60 to 75 minutes.

**Results:**

indicated that statistically significant differences at 0.01 level were found between mean scores of both groups groups on the posttest of psychological vitality favoring the experimental group. Also, statistically significant differences at 0.01 level were detected between the pre-test and post-test (experimental group) mean scores favoring the post-test. Taken together those findings confirm the effectiveness of the counseling program in improving psychological vitality Posttest and follow up test did not significantly differ which prove the continuity of program effectiveness.

**Conclusions:**

To conclude, motivational counseling plays an important role in enhancing psychological vitality of teachers. Further research might use the program in alleviating other psychological disorders.

**Disclosure:**

No significant relationships.

